# Developing a Measure to Assess General Knowledge for Coeliac Disease

**DOI:** 10.1111/hex.70623

**Published:** 2026-03-01

**Authors:** Tiffany Lavis, K. Ann Horsburgh, Jacqueline F. Gould, Elizabeth Bingham Thomas

**Affiliations:** ^1^ School of Psychology, Faculty of Health and Medical Sciences, The University of Adelaide Adelaide South Australia Australia; ^2^ School of Psychology, College of Education, Behavioural and Social Sciences, Adelaide University Adelaide South Australia Australia; ^3^ Department of Anthropology Florida State University Tallahassee Florida USA; ^4^ Native American and Indigenous Studies Center, Florida State University Tallahassee Florida USA; ^5^ School of Geography, Archaeology and Environmental Studies, University of the Witwatersrand, Wits South Africa; ^6^ SAHMRI Women and Kids, South Australian Health and Medical Research Institute North Adelaide South Australia Australia; ^7^ Department of Anthropology Brigham Young University Provo Utah USA

**Keywords:** assessment, autoimmune, celiac disease, coeliac disease, gluten, measurement development, symptom awareness

## Abstract

**Background:**

The importance of establishing knowledge about coeliac disease relates to the increasing incidence of symptomatic individuals who are undiagnosed.

**Objectives:**

This study aimed to develop and pilot a tool that could be used to measure general knowledge regarding coeliac disease.

**Design:**

A questionnaire was developed using 41 questions identified from an existing 300 published questions, to assess knowledge of symptoms, diagnosis, and management of coeliac disease (such as appropriate food preparation).

**Methods:**

Consenting participants completed the pilot questionnaire online (*N* = 359). Attrition analyses were conducted to determine whether there were any significant differences between those who completed the questionnaire (*n* = 284) and participants who left the study. Assessment of the questionnaire occurred in two stages. Initially, internal consistency, correlation, and exploratory factor analyses were conducted on the original 41 questions. Analyses were then repeated for the revised 25 questions, after low‐performing questions were removed.

**Results:**

Analyses of 284 participant responses indicated that low‐performing questions could be removed to consolidate the questions into a single, streamlined scale with 25 questions. Three sub‐scales were identified in the final questionnaire: basic knowledge, symptoms, and applied knowledge.

**Conclusions:**

The 41‐question measure effectively distinguishes low‐ and high‐levels of knowledge of coeliac disease; however, a streamlined scale of 25 questions can reduce completion time and attrition while maintaining validity. This questionnaire can be used for robust comparisons of knowledge between different populations. This questionnaire also offers a useful tool for identifying specific knowledge gaps, to guide efforts to raise awareness of coeliac disease in the general public, as well as for specific professions, to improve time to diagnosis, as well as food safety.

**Patient or Public Contribution:**

One of the authors is the mother of teenage children with coeliac disease. Another author was diagnosed with coeliac disease at the age of 27 years. Another author developed coeliac disease at age 16, after which it took her 19 years to achieve diagnosis. The other author does not have coeliac disease or an immediate family member with coeliac disease. The research team therefore has a productive combination of insiders and outsiders.

## 1 Introduction

Coeliac disease is an incurable autoimmune condition that results in an inappropriate immune response to the protein gluten. It is widely reported that approximately 80% of individuals with coeliac disease remain undiagnosed (see, e.g., [1, 2, 3]). Concurrently, there is a reported increase in the incidence of coeliac disease, with estimates suggesting prevalence rates between 1.3% and 1.8% of the general population (see., e.g., [4]). There are more than 250 known symptoms associated with coeliac disease although symptoms are highly variable between patients and therefore not always attributed to coeliac disease by patients or medical professionals [5]. Further, there is a proportion of individuals with coeliac disease for whom there are no obvious symptoms, known as silent coeliac disease (see, e.g., [4, 6]), and the fact that coeliac disease can develop at any age potentially adds to the complexity of diagnosis [5].

A lack of awareness and understanding of coeliac disease has the potential to contribute to individuals missing out on the necessary diagnosis and management of the disease. This is problematic both for the management of coeliac disease symptoms and prevention of long‐term consequences associated with unmanaged coeliac disease (e.g., increased risk of heart disease, cancer, infertility, diabetes, multiple sclerosis [7, 8, 9]). The only available treatment for coeliac disease is the maintenance of a life‐long gluten‐free diet (GFD). It is therefore necessary for not only the patient to receive comprehensive education about potential sources of gluten, but for everyone who makes food or shares kitchen equipment with a person with coeliac disease to receive the same information. Management of coeliac disease, then, is a fundamentally biocultural phenomenon (see [10]) in which a patient's biological status influences and is influenced by the social and cultural landscape in which they find themselves. Of relevance are the awareness held by the public, and the extent to which they are willing to act on that information to keep patients with coeliac disease safe. This highlights the importance of establishing current knowledge about coeliac disease, including knowledge gaps that can inform efforts to improve the diagnosis and management of coeliac disease.

### 1.1 Current Assessment Tools

Hall et al. [11] conducted a systematic review to identify written assessment tools that assess knowledge related to coeliac disease. The aim of their research was to identify gaps in understanding for individuals with diagnosed coeliac disease to tailor support and education [11]. Although the aim of the review focused on a subset of the population (i.e., those already diagnosed with coeliac disease), the overriding purpose of this review serves to inform knowledge more broadly. The authors identified a total of 25 studies for inclusion in the systematic review, and a total of nine knowledge domains emerged from analyses:

- general coeliac disease (CD) knowledge
- management of CD
- identifying gluten in the diet and as ingredients
- food labelling and legislation
- nutrients and a GFD
- food handling practices and training
- eating out
- medicines, health, and beauty; and
- diagnosing CD [11].

Collation of the questions from the 25 studies identified would canvass a breadth of knowledge through the use of the existing assessment tools. However, summation of the questions which explored knowledge and gluten‐containing food lists would result in a tool which exceeds 300 questions (see [11], Table 2). Although previous research has considered knowledge of coeliac disease, the approach has generally been piecemeal, and a consistent approach to establishing knowledge is lacking. Often, the research purposes have, understandably, considered specific populations (e.g., medical professionals; chefs), however even within these more targeted studies there have been variations in the questions used to establish knowledge (see, e.g., [12, 13]). The current study seeks to establish whether there is a streamlined question set that more effectively measures general knowledge associated with coeliac disease, to assist with measuring general awareness of coeliac disease, comparing awareness across populations, and evaluating educational or public health efforts raising awareness of coeliac disease.

## 2 Methods

### 2.1 Research Aims

The current study sought to establish a tool that could be used to identify general knowledge for coeliac disease.

### 2.2 Sample

Participants were undergraduate and postgraduate university students from a leading research‐intensive Australian university in a city of approximately 1.4 million people. The data were collected as part of a pilot study from 15 August 2025 to 9 September 2025. The inclusion criteria required participants to be at least 18 years of age, reside in Australia, and possess an understanding of English. A total of 359 participants were recruited for the study. One participant was removed for not meeting age requirements, and a total of 74 participants were removed because the pattern of missing data would not allow for the statistical analyses required (see, e.g., [14]). There were 284 participants who completed all the scale items, as either volunteers (*n* = 216) or in exchange for course credit (*n* = 68). The final sample included participants aged between 18 and 71, with an average age of 22.27 years (*Mdn* = 21.00; SD = 6.47). The participants were recruited as volunteers through posters that advertised the project on university noticeboards, and through messages posted by course conveners on course information pages. Disciplines that were approached to advertise the project included: Business; Dental Surgery; Engineering; Food and Nutrition Science; Health Service Management; Health and Medical Sciences; Medical Studies; Medicine; Psychology; Law; Nursing; Occupational Therapy; Oral Health; Speech Pathology; and Surgery. Volunteers were invited to enter a draw to win one of three $50 (Coles/Myer) vouchers, for taking the time to complete the study. Additionally, first‐year psychology students were invited to participate in exchange for course credit. Participants were also invited to forward the study to their peers through a study link address.

### 2.3 Measures

The researchers reviewed the 300 questions from the 25 studies identified by Hall et al. [11] and eliminated repetitive questions. The questionnaire for the current study included a subset of the original questions (see Supporting Information S1: Supporting Materials Table 1, for the shortlisted questions which were subsequently omitted, and the reasons for omission), focusing on those questions which address knowledge, diagnosis, and management of coeliac disease for individuals who have not been diagnosed with coeliac disease, as well as aspects of food knowledge. These questions were extracted from those identified by Hall et al. [11] which were predominantly categorised under “general CD knowledge”, "identifying gluten in diet and ingredients”, and “management”, with some crossover into “food‐handling practices and training” and “food labelling and legislation”. The current study did not seek to include the remaining knowledge domains outlined by Hall et al. [11], such as “diagnosing CD”; “eating out”; “medicines, health, and beauty”; and “nutrients and a GFD”. A total of 41 questions constituted the questionnaire for the current study (see Table 1). Questions were therefore omitted if there was an assumption in the question that the individual had a diagnosis of coeliac disease. Questions were also excluded if they were aimed at carers of individuals with coeliac disease (e.g., [15, 16, 17, 18, 19, 20, 21]). Supporting Information S1: Supporting Materials Table 1 includes the specific details of individually removed items and the rationale for removal. Additionally, questions were excluded where the primary focus was on knowledge in a professional capacity (e.g., dentists; gastroenterologists; staff of prepared food establishments [12, 22, 23, 24, 25, 26, 27, 28, 29, 30]). However, questions were retained where possible (i.e., they could be applied more generally, to assess knowledge outside of a specific professional population).

As shown in Table 1, the questionnaire for the current study included nine questions that considered basic information about coeliac disease. There were five questions that explored symptoms commonly associated with coeliac disease, and a total of seven questions that considered management of coeliac disease. Finally, there were 23 questions which considered participants' applied knowledge, including awareness of foods considered to be safe for coeliac disease and knowledge of cross‐contamination.

**Table 1:** Questions included in the assessment tool and original publication details.

Information covered by the questions	Publication	Number of questions
Basic knowledge: awareness of coeliac disease, prevalence, cause, and knowledge of gluten	Alorayyidh et al. [31] Kozhakhmetova et al. [32] Meyer et al. [33]^a^ Simpson et al. [13]	3 2 1 3
Symptoms: including associated diseases	Alorayyidh et al. [31] Kozhakhmetova et al. [32]	1 3
Management: chronicity, gluten‐free diet, professional consultation	Alorayyidh et al. [31] Kozhakhmetova et al. [32]^a^ Uršulin‐Trstenjak et al. [34]^a^	3 1 1
Applied Knowledge: presence of gluten, which foods are safe, cross‐contamination	Meyer et al. [33]^a^ Silvester et al. [35] Zhou et al. [36] Zhou et al. [36]^a^	1 1 12 9

^a^ Adapted for the current study.

### 2.4 Procedure

The study received ethics approval from The University of Adelaide Human Research Ethics Committee prior to recruitment. Participants were directed to the online questionnaire via a Qualtrics link. They were provided with information about the study and proceeded if they consented to participate. Along with questions about coeliac disease (Table 1), demographic questions were included for the purposes of identifying the sample characteristics (i.e., age, gender, marital status, occupational status, income, nationality, education). Additionally, participants were asked to specify if they knew anyone with coeliac disease (i.e., self; spouse/partner; child; parent; sibling; friend; coworker; other acquaintance), and if they cooked for them, to assist in establishing pre‐existing knowledge.

### 2.5 Analyses

Data were first recoded according to conventions within the previous literature (e.g., [31, 32, 35, 36]). Where scoring details were not provided, the questionnaire items were coded as binary variables (correct/incorrect). Analyses were selected on the basis of established statistical protocols associated with the data. The majority of data analyses were conducted using IBM SPSS Statistics Version 31. JASP Version 0.95.1 was used to conduct exploratory factor analyses (EFA) given that SPSS is unable to analyse ordinal data for EFA.

Analyses for attrition were conducted using *χ* ^2^ (see Supporting Information S1: Supporting Materials). Binary data that met the assumptions were interpreted using Pearson's *χ* ^2^. Where the assumptions were violated, binary data analyses were interpreted using Yates' Correction for Continuity. Additionally, categorical data that violated the assumptions were analysed and interpreted using Monte Carlo methods.

Internal consistency for the overall scale was calculated using a common reliability measure for survey design (i.e., Cronbach's *α*). Cronbach's *α* enables binary and continuous variables to be analysed within the same scale and was therefore appropriate for the current study. Point‐biserial correlations were calculated for each of the dichotomous scale items, as is appropriate to measure correlations between dichotomous and continuous variables. For EFA, the coefficients were set at 0.4 to comply with the recommendations of Tabachnick and Fidell [37], who suggested an inspection of the correlation matrix for evidence of coefficients greater than 0.3. A scree plot was used to identify the presence of components with eigenvalues exceeding 1. An oblique rotation method (oblimin) was used to account for correlation between questions. Polychoric/tetrachoric correlation matrices were used given that the data consisted of dichotomous items. The factoring method was principal axis factoring.

## 3 Results

### 3.1 Attrition

As noted above, the final sample (*n* = 284) used for analyses was a subset of the participant group who began the questionnaire (*N* = 359). The attrition across the questionnaire (21%, to completion) is a noteworthy aspect of this study, although characteristics were similar to participants who completed the study. Participants included 77% female, 20% male, 2% non‐binary, 0.4% transgender, and 0.4% “other” and took a median of 14.88 min to complete the entire questionnaire (i.e., demographic questions as well as the 41 questions about coeliac disease). Each of the questions was analysed to determine whether there was a significant difference between the participants who completed the full questionnaire and those who did not complete the questionnaire (see Supporting Information S1: Supporting Materials Table 2). Results indicated there was not a pattern to suggest a meaningful difference between completers and those who did not complete the questionnaire.

### 3.2 Internal Consistency

The full scale administered consisted of 41 questions about coeliac disease, which, when coded into binary (correct/incorrect) items, resulted in a total of 94 items. For example, the symptoms and signs that indicate the presence of coeliac disease in adults [32] is a single question that has 10 statements which were scored to indicate binary (correct/incorrect) knowledge. The Cronbach *α* coefficient for the full scale for the current study was calculated (*α* = 0.91, *N* = 94).

### 3.3 Factor Analysis

Exploratory factor analysis of the 41 questions (which included 94 items) was conducted to determine whether there were underlying factors observed within the overall scale for the current study. The data were ordinal and therefore polychoric correlations were conducted to enable factor analysis. The individual Measures of Sampling Adequacy (MSA) ranged from moderate to good (0.4 to > 0.07) with a number of exceptions which indicate the items might not be suited to factor extraction (i.e., those below 0.4). The Kaiser‐Meyer‐Olkin Measure of Sampling Adequacy (KMO) value was 0.708 (i.e., above 0.6) and Bartlett's Test of Sphericity was significant, *χ*²(∞) = 4371.00, *p* < 0.001, indicting appropriateness of the analyses. Visual inspection the scree plot indicated that a three‐factor solution existed, which explains 32.1% of the variance, which is acceptable. Table 2 shows the items included for each factor and the associated loadings.

**Table 2:** Factor items and loadings.

Factor Items	Loading
Factor 1	
Which of the following diseases can be associated with coeliac disease?	
*Osteopenia, osteoporosis*	0.789
*Herpetiformis dermatitis, psoriasis*	0.760
*Down syndrome, Turner syndrome*	0.759
*Immunoglobulin A deficiency*	0.727
*Autoimmune thyroiditis*	0.695
*Hypoplasia of tooth enamel*	0.678
*Delayed sexual development in children*	0.652
*Type 1 diabetes*	0.642
*Infertility*	0.637
*Peripheral neuropathy, ataxia, epilepsy*	0.636
*Autoimmune gastritis (pernicious anemia)*	0.625
*Recurrent aphthous stomatitis*	0.610
For what symptoms and signs can you suspect the presence of coeliac disease in an adult?	
*Osteoporosis*	0.754
*Elevated hepatic ALT and AST for unknown reasons*	0.688
*Short stature*	0.646
*Iron deficiency anaemia for unknown reasons*	0.645
*Weight deficiency*	0.638
*The presence of chronic fatigue syndrome*	0.587
*Frequent abdominal pain and bloating*	0.558
*Chronic diarrhea or constipation*	0.537
For what symptoms and signs can you suspect the presence of coeliac disease in a child?	
*Short stature*	0.701
*Iron deficiency anemia for unknown reasons*	0.696
*Frequent colds*	0.651
*Weight deficiency, decreased muscle mass*	0.643
*Irritability, tearfulness*	0.592
*Poor appetite*	0.578
*Vomiting*	0.532
*Big belly*	0.514
*Frequent abdominal pain*	0.512
*Sometimes no apparent symptoms*	0.473
*Chronic diarrhea or constipation*	0.454
Please decide if the following foods are Foods Allowed, Foods to Question, or Foods Not Allowed in a gluten‐free diet.	
*Buckwheat*	0.512
Coeliac disease is a genetic disease	0.506
Coeliac disease is an auto‐immune disease	0.402
Factor 2	
If you accidentally put croutons in the salad, to make it safe for the person with coeliac disease, would you:	0.747
A grill is being used to cook seasoned chicken breast. The regular seasoning recipe has gluten. You need to cook a chicken breast without seasoning for a patient on a gluten‐free diet. How would you cook this chicken?	0.745
If you were making a meal for a coworker with coeliac disease, which of the following would you regard as essential, nice but non‐essential, unnecessary in preparing their food.	
*Avoid cutting a gluten‐free sandwich with a knife you used to cut an ordinary sandwich*	0.726
*Use a deep fryer that has is used only for gluten‐free foods*	0.662
*Avoid cooking the gluten‐free pasta in the water that was used to cook the ordinary pasta*	0.589
*Wash your hands or change your gloves after touching gluten‐containing food*	0.588
*Avoid using an ordinary toaster to toast gluten‐free bread*	‐0.520
*Opening a new container of peanut butter*	0.465
It is safe to use the same gloves to touch gluten‐containing and gluten‐free food items because the amount of cross‐contamination from this practice is so small that no harm will happen to someone on a gluten‐free diet.	0.705
A frying pan is used to make breaded fish. If gluten‐free breaded fish is to be made later, what would be the correct strategy?	0.695
If you were making a burger for a person with coeliac disease and accidentally put a gluten‐free beef burger patty, lettuce, tomato, pickles and tomato sauce on an ordinary burger bun, would you:	0.668
Cheesecake is being served for dessert at dinner. Which of the following would you tell a patient on gluten‐free diet?	0.634
Pizzas were made for lunch. There are regular pizzas and gluten‐free pizzas. What is the correct strategy to cut the slices?	0.631
Spaghetti sauce is being added while cooking a gluten‐free meal. It is a famous imported brand. The ingredient list on the bottle mentions protein flour, but no gluten‐containing grains. Which of the following is the correct strategy?	0.568
You are cutting up slices of bread (both gluten‐containing and gluten‐free bread) on a cutting board. What is the correct strategy?	0.547
While making a gluten‐free meal, it is safe to use boiling water for gluten‐free pasta if it has already been used to cook wheat pasta as long as all visible bits of wheat pasta are carefully removed by running the water through a sieve/colander.	0.516
After cooking a beef burger patty and placing it on a gluten‐free bun for a person with coeliac disease, you discover that someone had just finished using the same frying pan to cook a toasted cheese sandwich. Do you:	0.458
A person with coeliac disease asks for salad dressing for their salad. You have only Italian salad dressing. You read the label that lists the following:	0.449
Ice cream is being served to a person on a gluten‐free diet. Which is the correct statement?	0.440
For what symptoms and signs can you suspect the presence of coeliac disease in an adult?	
*No apparent symptoms*	0.425
Factor 3	
Have you heard of coeliac disease?	0.707
What is gluten?	0.544
The gluten‐free diet is (*a lifelong diet*)	0.452
If you were making a meal for a coworker with coeliac disease, which of the following would you regard as essential, nice but non‐essential, unnecessary in preparing their food.	
*Keep all cheese separate from the food preparation area so it does not come in contact with gluten‐free food*	‐0.417
Gluten is present in which of the following?	0.412

Factor 1 reflects the symptoms associated with coeliac disease, and accounts for 16.6% of the total variance, with factors loading from 0.402 to 0.789. Factor 2 reflects the applied knowledge of coeliac disease, and accounts for 9.9% of the total variance, with factors loading from 0.425 to 0.747. Factor 3 reflects knowledge about the role of gluten in coeliac disease, and accounts for 5.6% of the total variance, with factors loading from 0.412 to 0.707. There were 35 (of the 94) items that did not load significantly onto the underlying factors (see Table 3).

**Table 3:** Factor items and loadings for items excluded from factors.

Items	Loading
Coeliac disease is (*chronic*)	0.581
The available treatment for coeliac disease	0.776
The affected part of the body from gluten	0.832
I don't need to see a doctor or a registered dietitian if I'm following the gluten‐free diet	0.912
Please decide if the following foods are Foods Allowed, Foods to Question, or Foods Not Allowed in a gluten‐free diet.	
*Cocoa*	0.984
*Rice crisp cereal*	0.961
*Soy sauce*	0.984
*Milk*	0.826
*Modified corn starch*	0.843
*Spelt*	0.904
*Egg noodles*	0.966
*Oatmeal*	0.992
*Malt vinegar*	0.856
*Croutons*	0.981
*Flavored yoghurt*	0.904
*Sausages*	0.826
*Imitation seafood*	0.845
*Balsamic vinegar*	0.968
*Chickpea flour*	0.850
*Glutinous rice*	0.862
For what symptoms and signs can you suspect the presence of coeliac disease in an adult?	
*Presence of irritable bowel syndrome*	0.755
What is coeliac disease?	0.739
What causes coeliac disease?	0.857
Is it recommended that close relatives of patients with coeliac disease be examined for coeliac disease?	0.861
Can coeliac disease be cured?	0.674
Gluten belongs to which of the following nutrient groups?	0.943
All fresh fruits are safe to eat for someone on a gluten‐free diet.	0.946
If you were making a meal for a coworker with coeliac disease, which of the following would you regard as essential, nice but non‐essential, unnecessary in preparing their food.	
*Make the food in a dedicated kitchen in which you prepare only gluten‐free food*	0.914
You are buying tomato sauce. How can you find out if it contains gluten?	0.861
A person on a gluten‐free diet wants to have a muffin. There is some pure millet flour and some oat flour in the kitchen. Which flour(s) are gluten‐free and safe to use to make the muffin?	0.928
A toaster oven in the kitchen is used to toast slices of bread for breakfast. Which of the following is the safest method for toasting gluten‐free bread?	0.884
Quinoa is for dinner today instead of rice. Which of the following is true?	0.918
Soy sauce needs to be added to a gluten‐free dish. Which of the following is the right strategy?	0.800
How many people do you think are affected by coeliac disease?	0.956
How many people do you think are affected by peanut allergy?	0.967

### 3.4 Point‐Biserial Correlations

Point‐biserial correlations were conducted to examine the relationship between each dichotomous scale item (coded as correct/incorrect) and the continuous scale total. The correlations were conducted for each of the 41 questions (i.e., 94 items; see Supporting Information S1: Supporting Materials Tables 3–7). Correlations were considered for two pre‐existing measures: *Assessment of Knowledge on Coeliac Disease* (AKCD [31]), and *Gluten‐Free Diet Knowledge Scale* (GFD‐KS [35]). Additionally, correlations were considered for questions from previous research studies.

A statistically significant relationship was identified between the full scale used in the current study and each of the individual scale items for the AKCD [31], although the correlations were low to medium. This indicates the items that form the AKCD provide some discrimination between participants who score high and those participants who score low on the full scale, although these items are not as strong as might be anticipated for an existing measure of knowledge. Similarly, a statistically significant relationship was identified between the full scale used in the current study and nine of the 13 individual scale items for the GFD‐KS [35], although the correlations were low to medium. Additionally, there were four items (i.e., *Cocoa, p* = 0.06; *Croutons, p* = 0.71; *Balsamic vinegar, p* = 0.23; *Oatmeal, p* = 0.18) which were non‐significant, indicating no statistically meaningful relationship with the overall scale score for the current study. There were six questions included in the current study from the research of Kozhakhmetova et al. [32]. Point‐biserial correlations were conducted to examine the relationship between these questions (coded as correct/incorrect) and the continuous scale total. One question (*What causes coeliac disease?*) was not significantly related to the scale total. However, all remaining questions included in the current study were significant, indicating discrimination between participants who score high and those participants who score low on the full scale.

There were 21 questions included in the current study from the research of Zhou et al. [36]. There were statistically significant relationships identified between the full scale used in the current study and seven of the eight items that formed the question, which considered actions when making a meal for a co‐worker with coeliac disease, which were low to medium correlations. The non‐significant (*p* = 0.31) relationship was for the question, *Keep all cheese separate from the food preparation area so it does not come in contact with gluten‐free food*. Additionally, there were low correlations for 11 of the remaining 20 questions, and medium correlations for the eight remaining correlations. There was one non‐significant (*p* = 0.12) correlation (i.e., *All fresh fruits are safe to eat by someone on a gluten‐free diet*).

Finally, there were six questions which came from various previous research studies, which were included for the current study [13, 33, 34]. Point‐biserial correlations were conducted to examine the relationship between these questions (coded as correct/incorrect) and the continuous scale total. There were two medium and three low significant correlations. Perhaps not surprisingly, there was a non‐significant (*p* = 0.09) relationship for the question, *How many people do you think are affected by peanut allergy?*

### 3.5 Adjusted Full Scale

Based on the above analyses, questions were reviewed to determine whether to include them in the final questionnaire. Perhaps surprisingly, some questions which form part of existing measures (e.g., AKCD) were identified to increase internal consistency if removed (i.e., *Coeliac disease is*; *The available treatment for coeliac disease*; *The affected part of the body from gluten*; *Need to see a doctor or registered dietician if following a gluten‐free diet*). However, there were indications that the overall measure was reliable, with these questions included. Therefore, the full measure was retained for the revised questionnaire in the current study.

However, the above analyses indicated appropriate removal of questions from the full scale to improve reliability. First, the question that asked participants about the cause of coeliac disease [32] was identified as one of the questions that, if removed, would increase internal consistency. The cause of coeliac disease was also identified as lacking connection to the underlying factors and the overall scale total. Two questions which assessed prevalence of coeliac disease [13], and comparison with prevalence of peanut allergy [13], were identified as lacking connection to the overall scale total, and were identified to increase internal consistency if removed. These three questions were removed from the full scale.

Three items which considered whether something was essential/nice but non‐essential/unnecessary when preparing a meal for a co‐worker [36] were identified to increase internal consistency if removed (i.e., *Keep all cheese separate from the food preparation area so it does not come in contact with gluten free food*; *Make the food in a dedicated kitchen in which you prepare only gluten‐free food*; *Avoid using an ordinary toaster to toast gluten‐free bread*). Although these items were recognised as assessing important aspects of food preparation, it was acknowledged that the questions may be unclear, especially where recommendations may vary (e.g., research has indicated guidelines for using a toaster [38]). These items were removed from the full scale. A further question [36] was identified to increase internal consistency if removed (i.e., *Gluten belongs to which of the following nutrient groups?*), and was therefore removed.

One aspect of the question which explored symptoms and signs for adults which indicate coeliac disease [32] was noted to improve reliability if removed (i.e., presence of IBS). This suggests further exploration of the context for IBS would be prudent. However, a decision was made to retain this aspect of the question for the full scale, since all other components of both the adult and child questions were retained. Conversely, there were some questions which, on reflection, lack clarity, and therefore not surprisingly were identified as non‐significantly correlated to the full scale or would improve reliability if removed (i.e., *can coeliac disease be cured*; *how to find out if tomato sauce is gluten‐free; whether types of flour and quinoa are gluten‐free; whether soy sauce is gluten‐free; whether all fresh fruit is gluten‐free; and whether it is safe to use a toaster*). It is noted that some of these questions are unlikely to be universally consistent, and these questions are likely to depend on labelling laws and production methods. Therefore, these questions were removed from the full scale.

Interestingly, all except one item (i.e., buckwheat) on the GFD‐KS [35] were identified to improve reliability if removed (i.e., *cocoa, croutons, balsamic vinegar, soy sauce, egg noodles, oatmeal*) or were otherwise not significantly discriminant. This indicates this approach was not particularly useful when attempting to determine general knowledge associated with coeliac disease. These questions were therefore removed from the full scale, including the item related to buckwheat.

Finally, there were questions identified that would improve reliability if removed; however, these questions were arguably important in the context of the current study. For example, *what is coeliac disease*, and *the need for family members to be examined for coeliac disease*. These questions are thought to form an important aspect of the questionnaire and were therefore retained.

#### 3.5.1 Adjusted Full Scale Analyses

The Cronbach *α* coefficient for the adjusted full scale was calculated (*α* = 0.92, *N* = 65). Exploratory factor analysis for the adjusted scale again indicated a three‐factor solution based on visual inspection the scree plot, which explains 46.1% of the variance; which is acceptable and within expectations for a psychological scale. The exploratory factor analysis indicated the KMO value was 0.737, and Bartlett's Test of Sphericity was significant, *χ*²(1711) = 130 242.24, *p* < 0.001, indicting appropriateness of the analyses. The MSA for individual items were above 0.5, suggesting items were suited to factor analysis. Factor 1 reflects symptoms associated with coeliac disease, which explains 29.4% of the total variance, with factors loading from 0.42 to 0.76. Factor 2 reflects applied knowledge for coeliac disease, which explains 11.5% of the total variance, with factors loading from 0.41 to 0.80. Factor 3 reflects the basic knowledge associated with coeliac disease (whether the participant has heard of coeliac disease, what coeliac disease is, what is gluten), which explains 5.2% of the total variance, with factors loading from 0.42 to. 74. There were three items which did not load significantly onto the underlying factors (i.e., *What is coeliac disease? Is it recommended that close relatives of patients with coeliac disease be examined for coeliac disease? Gluten is present in which of the following?*). Additionally, the *presence of IBS* item retained from Kozhakhmetova et al. [32] did not load significantly onto the underlying factors.

Although statistical analyses indicated three factors (i.e., basic knowledge, symptoms, applied knowledge) there were some loadings which were nonsensical (e.g., the presence of no adult symptoms within the applied knowledge factor). Therefore, expert judgment was applied to formulate the 25 questions (i.e., 65 items) into the three factors (see Table 4), retaining all items. The Cronbach *α* coefficient was calculated for each of the sub‐scales: *Basic Knowledge* (8 questions) (*α* = 0.55, *N* = 8 items), *Symptoms* (3 questions) (*α* = 0.93, *N* = 36 items), and *Applied Knowledge* (14 questions) (*α* = 0.76, *N* = 21 items).

**Table 4:** Retained items for the 25‐question coeliac disease knowledge scale.

Question	Response options		
1. Have you heard of coeliac disease?	1 = Yes 0 = No		
2. What is coeliac disease?	0 = Allergic disease 1 = Autoimmune disease 0 = Infectious disease 0 = Large bowel disorder 0 = Genetic disorder, gene mutation leads to disease in 100% of mutation carriers		
3. For what symptoms and signs can you suspect the presence of coeliac disease in an adult?	a0 = Adults do not have coeliac disease, it is a childhood disease 1 = Chronic diarrhea or constipation 1 = Weight deficiency 1 = Iron deficiency anaemia for unknown reasons 1 = Frequent abdominal pain and bloating 1 = Short stature 1 = Osteoporosis 1 = Presence of irritable bowel syndrome 1 = The presence of chronic fatigue syndrome 1 = Elevated hepatic ALT and AST for unknown reasons 1 = No apparent symptoms. 0 = I do not know		
4. For what symptoms and signs can you suspect the presence of coeliac disease in a child?	1 = Chronic diarrhea or constipation 1 = Frequent abdominal pain 1 = Big belly 1 = Vomiting 1 = Weight deficiency, decreased muscle mass 1 = Poor appetite 1 = Short stature 1 = Irritability, tearfulness 1 = Iron deficiency anemia for unknown reasons 1 = Frequent colds 1 = Sometimes no apparent symptoms 0 = I don't know		
5. Which of the following diseases can be associated with coeliac disease?	1 = Delayed sexual development in children 1 = Infertility 1 = Osteopenia, osteoporosis 1 = Immunoglobulin A deficiency 1 = Hypoplasia of tooth enamel 1 = Recurrent aphthous stomatitis 1 = Type 1 diabetes 1 = Autoimmune thyroiditis 1 = Autoimmune gastritis (pernicious anemia) 1 = Herpetiformis dermatitis, psoriasis 1 = Down syndrome, Turner syndrome 1 = Peripheral neuropathy, ataxia, epilepsy 0 = I don't know		
6. Is it recommended that close relatives of patients with coeliac disease be examined for coeliac disease?	1 = Yes 0 = No		
7. Coeliac disease is an auto‐immune disease	1 = Yes 0 = No 0 = I don't know		
8. Coeliac disease is a genetic disease	1 = Yes 0 = No 0 = I don't know		
9. The gluten‐free diet is	1 = A lifelong diet 0 = A temporary diet 0 = I don't know		
10. What is gluten?	1 = A protein in wheat 0 = An artificial food additive 0 = A flavour enhancer 0 = A food preservative		
11. Gluten is present in which of the following?	1 = Wheat, barley, rye 0 = Potato, rice, barley, wheat 0 = Wheat, potato, barley, millet 0 = Corn, rice, wheat, rye 0 = Barley, wheat, corn		
12. It is safe to use the same gloves to touch gluten‐containing and gluten‐free food items because the amount of cross‐contamination from this practice is so small that no harm will happen to someone on a gluten‐free diet.	0 = True 1 = False		
13. While making a gluten‐free meal, it is safe to use boiling water for gluten‐free pasta if it has already been used to cook wheat pasta, as long as all visible bits of wheat pasta are carefully removed by running the water through a sieve/colander.	0 = True 1 = False		
14. Cheesecake is being served for dessert at dinner. Which of the following would you tell a patient on gluten‐free diet?	1 = Do not eat the cheesecake. Offer a different dessert option that is safe. 0 = Eat only the top layer of the cheesecake, which is gluten‐free and toss out the gluten‐containing crust 0 = Eat the whole cheesecake as the amount of gluten in the crust is extremely low		
15. Spaghetti sauce is being added while cooking a gluten‐free meal. It is a famous imported brand. The ingredient list on the bottle mentions protein flour, but no gluten‐containing grains. Which of the following is the correct strategy?	0 = Use the sauce, as it is most likely gluten‐free 1 = Do not use the sauce in the meal 0 = Use only a very small amount of sauce, just in case it has traces of gluten		
16. A frying pan is used to make breaded fish. If gluten‐free breaded fish is to be made later, what would be the correct strategy?	0 = The same oil can be used as it is cost effective and risk of gluten contamination is extremely low 0 = All bits of breading should be removed carefully from the oil before frying gluten‐free fish 1 = The pan should be cleaned and fresh oil used		
17. A grill is being used to cook seasoned chicken breast. The regular seasoning recipe has gluten. You need to cook a chicken breast without seasoning for a patient on a gluten‐free diet. How would you cook this chicken?	0 = Use the same grill because the heat kills gluten, removing any risk of contamination 0 = Use the same grill after brushing it off because this will remove the gluten 1 = Cook the chicken in a clean pan because using the same grill is unsafe for someone on a gluten‐free diet		
18. Pizzas were made for lunch. There are regular pizzas and gluten‐free pizzas. What is the correct strategy to cut the slices?	0 = Use the same knife because it is the fastest and the risk of cross‐contamination is very low 0 = Use the same knife as long as you wipe the knife with a cloth in between pizzas 1 = Use only a separate, clean knife to cut the gluten‐free pizzas		
19. You are cutting up slices of bread (both gluten‐containing and gluten‐free bread) on a cutting board. What is the correct strategy?	0 = Use the same wooden cutting board for both types of bread. The crumbs are too small to cause any problems for patients on a gluten‐free diet. 0 = Use the same wooden cutting board as long as you wash it before cutting the gluten‐free bread 1 = Do not use the wooden cutting board for the gluten‐free bread because it is impossible to clean out very small pieces of gluten. You must have a dedicated cutting board		
20. A person with coeliac disease asks for salad dressing for their salad. You have only Italian salad dressing. You read the label that lists the following: Soybean oil, water, vinegar, dehydrated Romano cheese, extra virgin olive oil, salt, sugar, garlic powder, spices, anchovy paste (anchovies, salt), malted barley, citric acid, xanthan gum, dehydrated Worcestershire sauce (maltodextrin, vinegar, molasses, corn syrup, water, salt, caramel, garlic powder, sugar, spices, tamarind, flavour, sulphites). Is it safe to serve this salad dressing to the person?	0 = Yes 1 = No		
21. Ice cream is being served to a person on a gluten‐free diet. Which is the correct statement?	0 = Ice cream is safe because milk does not contain gluten 1 = Ice cream may contain gluten so the ingredient list should be checked on the container 0 = Ice cream is only unsafe if served on an ice cream cone		
22. If you accidentally put croutons in the salad, to make it safe for the person with coeliac disease, would you:	1 = make a new salad 0 = pick out and discard the croutons 0 = pick out and discard the croutons, and the pieces of lettuce they were sitting on 0 = not worry about it because there isn't enough gluten in a few croutons to cause a problem		
23. If you were making a burger for a person with coeliac disease and accidentally put a gluten‐free beef burger patty, lettuce, tomato, pickles and tomato sauce on an ordinary burger bun, would you:	0 = wipe the tomato sauce off the patty, put new lettuce, tomato, pickles and tomato sauce on a gluten‐free bun and add the patty 0 = move everything from the ordinary burger bun to a gluten‐free burger bun 1 = discard the already‐made burger, and start a new one to put on the gluten‐free bun		
24. After cooking a beef burger patty and placing it on a gluten‐free bun for a person with coeliac disease, you discover that someone had just finished using the same frying pan to cook a toasted cheese sandwich. Do you:	0 = not worry about it because there couldn't be enough gluten left in the pan to be a problem 0 = give the burger to someone else, wipe out the pan and start over 1 = wash the pan with hot, soapy water before cooking a new burger patty in it		
25. If you were making a meal for a coworker with coeliac disease, which of the following would you regard as essential, nice but non‐essential, unnecessary in preparing their food.	Essential	Nice but non‐essential	Unnecessary
Opening a new container of peanut butter	1	0	0
Keep all cheese separate from the food preparation area so it does not come in contact with gluten‐free food	1	0	0
Use a deep fryer that is used only for gluten‐free foods	1	0	0
Wash your hands or change your gloves after touching gluten‐containing food	1	0	0
Make the food in a dedicated kitchen in which you prepare only gluten‐free food	0	1	0
Avoid cutting a gluten‐free sandwich with a knife you used to cut an ordinary sandwich	1	0	0
Avoid using an ordinary toaster to toast gluten‐free bread	1	0	0
Avoid cooking the gluten‐free pasta in the water that was used to cook the ordinary pasta	1	0	0

^a^ Consistent with Kozhakhmetova et al. [32], this item is included in the survey; however, it does not form part of the scoring.

## 4 Discussion

The current study explored 41 questions in a full‐scale to measure general knowledge for coeliac disease, based on extraction from over 300 questions from previous assessment tools (see [11]). A number of questions were identified to discriminate between participants with good knowledge of coeliac disease and those with poorer knowledge. The 41 questions implemented here had a median time to completion of 15 min. By selectively removing low‐performing questions, we were able to consolidate the original 41 questions into a single, streamlined scale with 25 questions. We are hopeful that the reduced list will result in a median time to completion of closer to 10 min, thereby reducing the proportion of respondents who abandon the questionnaire part‐way through.

Exploratory factor analyses for both the original 41 questions and the streamlined 25 questions revealed three coherent dimensions, which together explained 46.1% of the variance in the 25‐question scale. The factors reflect basic knowledge of coeliac disease, knowledge of symptoms associated with coeliac disease, and applied knowledge (i.e., about safe food handling practices). Although the questions were broadly extracted from the domains identified by Hall et al. [11], the final questionnaire formulated through the current study canvasses only aspects of these nine domains, which could be further explored in future research. The 25‐question measure effectively distinguishes low‐ and high‐levels of knowledge of coeliac disease. However, it is acknowledged that the current study canvassed a subset of the general population (i.e., university students). The study aimed to target students from both health‐related and non‐health‐related disciplines, including both students who will form our future professions, coming into contact with patients who will present with coeliac symptoms, as well as students who will be peripheral to these professions. We know that students are more likely to have knowledge of coeliac disease than other subsets of the general population [39] so awareness in the broader population in Australia may be poorer. Future research should include more diverse populations to validate the questions identified in the current study and ensure the tool is predictive across different demographic groups.

The current 25‐question measure provides a useful tool that can guide information gathering, public health campaigns, and educational initiatives. This questionnaire could reasonably be used for tertiary students, such as to determine adequate coverage of coeliac disease in health‐related courses. For example, the questionnaire could be used to establish the basic knowledge held by students who are studying toward professions where individuals are likely to present with symptoms of coeliac disease. This could be used to guide curriculum development, providing more targeted knowledge across university studies. Similarly, as the questionnaire is validated across the general population, this will further inform how to raise awareness. Implementation of the questionnaire will identify knowledge gaps, indicate how to approach awareness campaigns, and whether a focus is needed on safe food handling or diagnosis. The 25‐question measure has the potential to be used to structure robust comparisons of knowledge between different populations that can identify target audiences for awareness campaigns. Additionally, it will be a useful tool for identifying specific knowledge gaps in the general public to shape educational campaigns. The success of such campaigns can then be measured by deploying the questionnaire before and after implementation. Greater awareness of coeliac disease symptoms, and the variety of presentations across individuals with coeliac disease, would contribute to increased appropriate diagnoses of symptomatic individuals. The experience of living with coeliac disease, once diagnosed, is fundamentally dependent on the availability of safe food, by the knowledge of people in the food sector, on the knowledge of people in the patient's social circle, and on the willingness of those people to engage in the kinds of labour essential to avoid cross‐contamination. Improvements in the knowledge base of the non‐coeliac population are likely to directly improve the quality of life of people with coeliac disease.

## Author Contributions

**Tiffany Lavis:** conceptualisation, data curation, formal analysis, investigation, methodology, project administration, writing – original draft, writing – review and editing. **Jacqueline F. Gould:** conceptualisation, methodology, writing – review and editing. **K. Ann Horsburgh:** conceptualisation, investigation, writing – original draft, writing – review and editing. **Elizabeth Bingham Thomas:** conceptualisation, methodology, writing – review and editing.

## Funding

The authors received no specific funding for this work.

## Ethics Statement

The study received ethics approval from The University of Adelaide Human Research Ethics Committee prior to data collection.

## Conflicts of Interest

The authors declare no conflicts of interest.

## Supporting information


**FIGURE 1:** Attrition across the questionnaire as indicated by participant drop‐out.

## Data Availability

The data that support the findings of this study are currently available from the corresponding author upon reasonable request.
