# Supplementary material for: Developing a Measure to Assess General Knowledge for Coeliac Disease
Source: Health Expect. 2026 Mar 1;29(2):e70623. doi: 10.1111/hex.70623 (PMC12949961; doi:10.1111/hex.70623)
Supplement: Supplementary file 1 — FIGURE 1: Attrition across the questionnaire as indicated by participant drop‐out. [file HEX-29-e70623-s001.docx]

**Supplementary Materials**

*Note. Minor adjustments have not been noted (e.g., where celiac disease or CD were changed to coeliac disease; where language was updated to English spelling).*

[**The 41-item survey used in the current study** 2](#_Toc220331263)

[**Questions shortlisted and omitted from the 41-item survey** 18](#_Toc220331264)

[Questions omitted from the 41-item survey and the reasons for omission. 18](#_Toc220331265)

[**Attrition analyses** 26](#_Toc220331266)

[**Analyses to compare participant survey completers and non-completers** 28](#_Toc220331267)

[Chi-square analyses to compare participant completers versus non-completers. 28](#_Toc220331268)

[**Correlations between individual items and the full 41-item scale** 40](#_Toc220331269)

[Point-biserial correlation results for the AKCD (Alorayyidh et al., 2023) questions included in the 41-item scale 40](#_Toc220331270)

[Point-biserial correlation results for the questions from Kozhakhmetova et al. (2022) included in the 41-item scale 41](#_Toc220331271)

[Point-biserial correlation results for the GFD-KS (Silvester et al., 2016) questions included in the 41-item scale 44](#_Toc220331272)

[Point-biserial correlation results for the questions from Zhou et al. (2022) included in the 41-item scale 45](#_Toc220331273)

[Point-biserial correlation results for the additional questions from previous research included in the 41-item scale 49](#_Toc220331274)

## **The 41-item survey used in the current study**

*Original publication details are included in () above the question set. Scoring details are included below the question text. The correct answer is in bold (where applicable).*

(*Simpson et al., 2011*)

1. Have you heard of coeliac disease?

- Yes
- No

*(Yes = 1; No = 0)*

2. How many people do you think are affected by coeliac disease?

- 1 in 10
- 1 in 50
- **1 in 100**
- 1 in200
- 1 in 500
- 1 in 1000

*(Correct answer = 1; incorrect answers = 0)*

3. How many people do you think are affected by peanut allergy?

- 1 in 10
- 1 in 50
- **1 in 100**
- 1 in200
- 1 in 500
- 1 in 1000

*(Correct answer = 1; incorrect answers = 0)*

(*Kozhakhmetova et al., 2022*)

4. What is coeliac disease?

- Allergic disease
- **Autoimmune disease**
- Infectious disease
- Large bowel disorder
- Genetic disorder, gene mutation leads to disease in 100% of mutation carriers

*(Correct answer = 1; incorrect answers = 0)*

5. What causes coeliac disease?

- Gluten intolerance
- Intolerance to dairy products
- Allergy
- **Gut dysbiosis**

*(Correct answer = 1; incorrect answers = 0)*

6. For what symptoms and signs can you suspect the presence of coeliac disease in an adult (tick all that apply)?

- Adults do not have coeliac disease, it is a childhood disease
- **Chronic diarrhea or constipation**
- **Weight deficiency**
- **Iron deficiency anaemia for unknown reasons**
- **Frequent abdominal pain and bloating**
- **Short stature**
- **Osteoporosis**
- **Presence of irritable bowel syndrome**
- **The presence of chronic fatigue syndrome**
- **Elevated hepatic ALT and AST for unknown reasons**
- **No apparent symptoms.**
- I do not know

*(Correct answers each = 1; incorrect answers = 0)*

7. For what symptoms and signs can you suspect the presence of coeliac disease in a child (tick all that apply)?

- **Chronic diarrhea or constipation**
- **Frequent abdominal pain**
- **Big belly**
- **Vomiting**
- **Weight deficiency, decreased muscle mass**
- **Poor appetite**
- **Short stature**
- **Irritability, tearfulness**
- **Iron deficiency anemia for unknown reasons**
- **Frequent colds**
- **Sometimes no apparent symptoms.**
- I do not know

*(Correct answers each = 1; incorrect answer = 0)*

8. Which of the following diseases can be associated with coeliac disease (tick all that apply)?

- **Delayed sexual development in children**
- **Infertility**
- **Osteopenia, osteoporosis**
- **Immunoglobulin A deficiency**
- **Hypoplasia of tooth enamel**
- **Recurrent aphthous stomatitis**
- **Type 1 diabetes**
- **Autoimmune thyroiditis**
- **Autoimmune gastritis (pernicious anemia)**
- **Herpetiformis dermatitis, psoriasis**
- **Down syndrome, Turner syndrome**
- **Peripheral neuropathy, ataxia, epilepsy**
- I don't know

*(Correct answers each = 1; incorrect answer = 0)*

(*Adapted from: Kozhakhmetova et al., 2022*)

9. Is it recommended that close relatives of patients with coeliac disease be examined for coeliac disease?

- **Yes**
- No

*(Correct answer = 1; incorrect answer = 0)*

(*Alorayyidh, et al., 2023*)

10. Coeliac disease is an auto-immune disease

- **Yes**
- No
- I don’t know

*(Correct answer = 1; incorrect answers = 0)*

11. Coeliac disease is

- **Chronic**
- Acute
- I don’t know

*(Correct answer = 1; incorrect answers = 0)*

12. Coeliac disease is a genetic disease

- **Yes**
- No
- I don’t know

*(Correct answer = 1; incorrect answers = 0)*

13. The available treatment for coeliac disease

- **Gluten-free-diet**
- Medications
- Gluten-free-diet and medications
- I don’t know

*(Correct answer = 1; incorrect answers = 0)*

14. The affected part of body from gluten

- Stomach
- **Small intestine**
- Large intestine

*(Correct answer = 1; incorrect answers = 0)*

15. The gluten-free diet is

- **A lifelong diet**
- A temporary diet
- I don’t know

*(Correct answer = 1; incorrect answers = 0)*

16. I don’t need to see a doctor or a registered dietitian if I‘m following the gluten free diet

- True
- **False**

*(Correct answer = 1; incorrect answer = 0)*

(*Adapted from:* *Uršulin-Trstenjak et al., 2019*)

17. Can coeliac disease be cured?

- Yes
- **No**
- I don’t know

*(Correct answer = 1; incorrect answers = 0)*

(*Adapted from: Meyer et al., 2004*)

18. What is gluten?

- **A protein in wheat**
- An artificial food additive
- A flavour enhancer
- A food preservative

*(Correct answer = 1; incorrect answers = 0)*

19. You are buying tomato sauce. How can you find out if it contains gluten?

- **I’m reading the ingredients on the bottle**
- I suppose that tomato sauce doesn’t usually contain gluten
- I only buy reduced sugar or salt tomato sauce!
- I’m reading the “list of gluten free food” or discover on the packaging the word gluten free

*(Correct answer = 1; incorrect answers = 0)*

(*Silvester et al., 2016*)

20. Please decide if the following foods are Foods Allowed, Foods to Question, or Foods Not Allowed in a gluten-free diet.

|  | Allowed | Foods to question | Not allowed |
| --- | --- | --- | --- |
| Cocoa | **⚫** | ⭘ | ⭘ |
| Malt vinegar | ⭘ | ⭘ | **⚫** |
| Croutons | ⭘ | **⚫** | ⭘ |
| Flavored yoghurt | ⭘ | **⚫** | ⭘ |
| Sausages | ⭘ | **⚫** | ⭘ |
| Imitation seafood | ⭘ | **⚫** | ⭘ |
| Balsamic vinegar | **⚫** | ⭘ | ⭘ |
| Chickpea flour | **⚫** | ⭘ | ⭘ |
| Glutinous rice | **⚫** | ⭘ | ⭘ |
| Rice crisp cereal | ⭘ | **⚫** | ⭘ |
| Soy sauce | ⭘ | **⚫** | ⭘ |
| Milk | **⚫** | ⭘ | ⭘ |
| Buckwheat | **⚫** | ⭘ | ⭘ |
| Modified corn starch | **⚫** | ⭘ | ⭘ |
| Spelt | ⭘ | ⭘ | **⚫** |
| Egg noodles | ⭘ | ⭘ | **⚫** |
| Oatmeal | ⭘ | **⚫** | ⭘ |

*(Correct answers each = 1; incorrect answers = 0)*

(*Zhou et al., 2022*)

21. Gluten belongs to which of the following nutrient groups?

- Carbohydrate
- **Protein**
- Fat
- Vitamins
- Minerals

*(Correct answer = 1; incorrect answers = 0)*

22. Gluten is present in which of the following?

- **Wheat, barley, rye**
- Potato, rice, barley, wheat
- Wheat, potato, barley, millet
- Corn, rice, wheat, rye
- Barley, wheat, corn

*(Correct answer = 1; incorrect answers = 0)*

23. All fresh fruits are safe to eat by someone on a gluten-free diet.

- **True**
- False

*(Correct answer = 1; incorrect answer = 0)*

24. It is safe to use the same gloves to touch gluten-containing and gluten-free food items because the amount of cross contamination from this practice is so small that no harm will happen to someone on a gluten-free diet.

- True
- **False**

*(Correct answer = 1; incorrect answer = 0)*

25. Soy sauce needs to be added to a gluten-free dish. Which of the following is the right strategy?

- Soy sauce can be safely added as soy is gluten free.
- No type of soy sauce should be used at all.
- **Check the ingredient list to determine if the soy sauce has gluten.**

*(Correct answer = 1; incorrect answers = 0)*

26. While making a gluten-free meal, it is safe to use boiling water for gluten-free pasta if it has been already used to cook wheat pasta as long as all visible bits of wheat pasta are carefully removed by running the water through a sieve/colander.

- True
- **False**

*(Correct answer = 1; incorrect answer = 0)*

27. Cheesecake is being served for dessert at dinner. Which of the following would you tell a patient on gluten free diet?

- **Do not eat the cheesecake. Offer a different desert option that is safe.**
- Eat only the top layer of the cheesecake which is gluten-free and toss out the gluten-containing crust
- Eat the whole cheesecake as the amount of gluten in the crust is extremely low.

*(Correct answer = 1; incorrect answers = 0)*

28. Spaghetti sauce is being added while cooking a gluten-free meal. It is a famous imported brand. The ingredient list on the bottle mentions protein flour, but no gluten-containing grains. Which of the following is the correct strategy?

- Use the sauce, as it is most likely gluten-free.
- **Do not use the sauce in the meal.**
- Use only a very small amount of sauce, just in case it has traces of gluten.

*(Correct answer = 1; incorrect answers = 0)*

29. A frying pan is used to make breaded fish. If gluten-free breaded fish is to be made later, what would be the correct strategy?

- The same oil can be used as it is cost effective and risk of gluten contamination is extremely low.
- All bits of breading should be removed carefully from the oil before frying gluten-free fish.
- **The pan should be cleaned and fresh oil used.**

*(Correct answer = 1; incorrect answers = 0)*

30. A grill is being used to cook seasoned chicken breast. The regular seasoning recipe has gluten. You need to cook a chicken breast without seasoning for a patient on a gluten-free diet. How would you cook this chicken?

- Use the same grill because the heat kills gluten, removing any risk of contamination.
- Use the same grill after brushing it off because this will remove the gluten.
- **Cook the chicken in a clean pan because using the same grill is unsafe for someone on a gluten-free diet.**

*(Correct answer = 1; incorrect answers = 0)*

31. Pizzas were made for lunch. There are regular pizzas and gluten-free pizzas. What is the correct strategy to cut the slices?

- Use the same knife because it is the fastest and the risk of cross contamination is very low.
- Use the same knife as long as you wipe the knife with a cloth in between pizzas.
- **Use only a separate, clean knife to cut the gluten-free pizzas.**

*(Correct answer = 1; incorrect answers = 0)*

32. You are cutting up slices of bread (both gluten-containing and gluten-free bread) on a cutting board. What is the correct strategy?

- Use the same wooden cutting board for both types of bread. The crumbs are too small to cause any problems for patients on a gluten-free diet.
- Use the same wooden cutting board as long as you wash it before cutting the gluten-free bread.
- **Do not use the wooden cutting board for the gluten-free bread because it is impossible to clean out very small pieces of gluten. You must have a dedicated cutting board.**

*(Correct answer = 1; incorrect answers = 0)*

(*Adapted from: Zhou et al., 2022*)

33. A person with coeliac disease asks for salad dressing for their salad. You have only Italian salad dressing. You read the label that lists the following:

Soybean oil, water, vinegar, dehydrated Romano cheese, extra virgin olive oil, salt, sugar, garlic powder, spices, anchovy paste (anchovies, salt), malted barley, citric acid, xanthan gum, dehydrated Worcestershire sauce (maltodextrin, vinegar, molasses, corn syrup, water, salt, caramel, garlic powder, sugar, spices, tamarind, flavour, sulphites).

It is safe to serve this salad dressing to the person?

- Yes
- **No**

*(Correct answer = 1; incorrect answer = 0)*

34. A person on a gluten-free diet wants to have a muffin. There is some pure millet flour and some oat flour in the kitchen. Which flour(s) are gluten free and safe to use to make the muffin?

- Both flours are safe to use
- **Only millet flour can be used safely**
- Only oat flour can be used safely
- Neither flour is safe to use

*(Correct answer = 1; incorrect answers = 0)*

35. Ice cream is being served to a person on a gluten-free diet. Which is the correct statement?

- Ice cream is safe because milk does not contain gluten.
- **Ice cream may contain gluten so the ingredient list should be checked on the container.**
- Ice cream is only unsafe if served on an ice cream cone.

*(Correct answer = 1; incorrect answers = 0)*

36. If you accidently put croutons in the salad, to make it safe for the person with coeliac disease, would you:

- - **make a new salad**
  - pick out and discard the croutons
  - pick out and discard the croutons, and the pieces of lettuce they were sitting on
  - not worry about it because there isn’t enough gluten in a few croutons to cause a problem

*(Correct answer = 1; incorrect answers = 0)*

37. A toaster oven in the kitchen is used to toast slices of bread for breakfast. Which of the following is the safest method for toasting gluten-free bread?

- Remove any crumbs carefully from the oven before putting in the gluten-free bread.
- **Put the slice of gluten-free bread in an enclosed, oven-safe container and put in the oven.**
- Put the gluten-free bread on an aluminum tray before placing in the oven.

*(Correct answer = 1; incorrect answers = 0)*

38. If you were making a burger for a person with coeliac disease and accidentally put a gluten-free beef burger patty, lettuce, tomato, pickles and tomato sauce on an ordinary burger bun, would you:

- wipe the tomato sauce off the patty, put new lettuce, tomato, pickles and tomato sauce on a gluten free bun and add the patty
- move everything from the ordinary burger bun to a gluten free burger bun
- **discard the already made burger, and start a new one to put on the gluten free bun**

*(Correct answer = 1; incorrect answers = 0)*

39. Quinoa is for dinner today instead of rice. Which of the following is true?

- Quinoa is always safe for someone on a gluten-free diet because it is a gluten-free grain.
- **Quinoa is safe for someone on a gluten-free diet only if the label states “gluten free” because this grain can be contaminated with other grains that have gluten.**
- Quinoa is never safe for someone on a gluten-free diet because it contains gluten.

*(Correct answer = 1; incorrect answers = 0)*

40. After cooking a beef burger patty and placing it on a gluten-free bun for a person with coeliac disease, you discover that someone had just finished using the same frying pan to cook a toasted cheese sandwich. Do you:

- - not worry about it because there couldn’t be enough gluten left in the pan to be a problem
  - give the burger to someone else, wipe out the pan and start over
  - **wash the pan with hot, soapy water before cooking a new burger patty in it**

*(Correct answer = 1; incorrect answers = 0)*

41. If you were making a meal for a coworker with coeliac disease, which of the following would you regard as essential, nice but non-essential, unnecessary in preparing their food.

|  | Essential | Nice but non-essential | Unnecessary |
| --- | --- | --- | --- |
| Opening a new container of peanut butter | **⚫** | ⭘ | ⭘ |
| Keep all cheese separate from the food preparation area so it does not come in contact with gluten free food | **⚫** | ⭘ | ⭘ |
| Use a deep fryer that is used only for gluten-free foods | **⚫** | ⭘ | ⭘ |
| Wash your hands or change your gloves after touching gluten-containing food | **⚫** | ⭘ | ⭘ |
| Make the food in a dedicated kitchen in which you prepare only gluten-free food | ⭘ | **⚫** | ⭘ |
| Avoid cutting a gluten free sandwich with a knife you used to cut an ordinary sandwich | **⚫** | ⭘ | ⭘ |
| Avoid using an ordinary toaster to toast gluten-free bread | **⚫** | ⭘ | ⭘ |
| Avoid cooking the gluten free pasta in the water that was used to cook the ordinary pasta | **⚫** | ⭘ | ⭘ |

*(Correct answers each = 1; incorrect answers = 0)*

## **Questions shortlisted and omitted from the 41-item survey**

**TABLE 1**

### Questions omitted from the 41-item survey and the reasons for omission.

| Reason for Omission | Questions |
| --- | --- |
| Irrelevant to aims of the assessment tool | Would you like to know more about celiac disease?ᵃ |
|  | If so, what information would you like to receive?ᵃ |
|  | Do you know what a gluten-free product logo looks like?ᵈ |
|  | Gluten-free products can be bought in any store?ᵈ |
| Specific to professional role | What examination do you prescribe if you suspect celiac disease in a patient (tick all that apply)?ᵃ |
|  | What examination is necessary to confirm the diagnosis of celiac disease (golden standard)?ᵃ |
|  | Do you advise close relatives of patients with celiac disease to be examined for celiac disease?ᵃ |
|  | The most important examination for the diagnosis of CD isᵇ |
|  | What medicines are surly gluten free, can be taken in undoubtedly?ᵇ |
|  |  |
| Not specific to coeliac disease knowledge | Have you heard of peanut allergy?ᶜ |
|  | Have you heard of Gluten sensitivity?ᶜ |
|  | How many times per week would you eat outside in a restaurant, at friends’ houses, take-out/cafeteria food?ᶜ |
| Clarity concerns | Can gluten be found as a covered ingredient in some products?ᵈ |
|  | All canned pudding is safe to eat by someone on a gluten-free diet.ᶠ |
|  | Which of the following is safe in a gluten-free diet?ᶠ |
| Question unable to be objectively measured | Do you know what celiac disease is?ᵈ |
|  | The public is not educated enough about how much gluten-free foods are harmful to people suffering from celiac disease?ᵈ |
|  | Do you prefer a gluten-free diet regardless of the fact that you do not suffer from celiac disease?ᵈ |
|  | How much do you know about CD?ᵉ |
|  | How do you think people with CD feel?ᵉ |
|  | Does a person with CD enjoy eating?ᵉ |
|  | What would you do if it was your birthday and a classmate had CD?ᵉ |
|  | When do people with CD develop symptoms?ᵉ |
|  | Which of the following are definitely safe for someone on a gluten-free diet?ᶠ |
|  | Which of the following is always gluten-free?ᶠ |
| Difficult to verify with consistency | All products labelled "organic" are also "gluten-free".ᶠ |
|  | All products labelled "wheat-free" are also "gluten-free".ᶠ |
|  | Which of the following are always safe for someone on a gluten-free diet?ᶠ |
|  | All dried beans and lentils are safe to eat for someone on a gluten-free diet. ᶠ |
|  | A patient with celiac disease requests corn flakes for breakfast. You read the cereal box and it lists 100% corn, sugar, salt and malt flavoring. Is it safe to serve these corn flakes to the patient?ᶠ |
|  | You have run out of the original gluten-free dessert option for a patient on a gluten-free diet. What would you substitute on the tray instead?ᶠ |
|  | A hospitalized patient on a gluten-free diet is allowed only liquids and no solid food. You would like to give them an Ensure drink. Is this drink allowed on a gluten-free diet? Pick the best answer.ᶠ |
|  | An elderly man on a gluten-free diet is admitted for surgery. At breakfast, a muffin made from wheat flour got served to him instead of a gluten-free one. You had served the meal and find out about this in the evening. What is the best next step?ᶠ |
| Other questions cover the content | What is the main treatment for celiac disease?ᵃ |
|  | Celiac disease is a disease that only affects children?ᵈ |
|  | Primary treatment is a gluten-free diet?ᵈ |
|  | Which cereals should not be consumed in celiac disease?ᵈ |
|  | Can a gluten-free diet completely eliminate the symptoms of celiac disease?ᵈ |
|  | Gluten intolerance is the same as celiac disease?ᵈ |
|  | Does anyone in your family suffer from celiac disease?ᵈ |
|  | Do you suffer from celiac disease?ᵈ |
|  | What is gluten?ᵈ |
|  | Which foods contain gluten?ᵈ |
|  | What compound in food is harmful to people with CD?ᵉ |
|  | Which of the following foods a person with CD cannot eat?ᵉ |
|  | From the following list of foods, which ones may contain gluten?ᵉ |
|  | Which of the following is a gluten-free grain?ᶠ |
|  | Glutinous rice flour is safe to eat by someone on a gluten-free diet.ᶠ |
|  | Which of the following should NOT be consumed by someone on a gluten-free diet?"ᶠ |
|  | A patient on a gluten-free diet is asking for a piece of fresh fruit for their lunch. Is it safe to give it to them?ᶠ |

ᵃ**(**Kozhakhmetova et al.**,** 2022)

ᵇ(Meyer et al., 2004)

ᶜ(Simpson et al., 2011)ᵈ(Uršulin-Trstenjak et al., 2019)

ᵉ(Vázquez-Polo, et al., 2024)

ᶠ(Zhou et al., 2022)

### **Attrition analyses**

Attrition analyses indicated there was not a significant age difference between the participant group which was retained (*M* = 22.27, *SD* = 6.47) and the participant group with unusable data (*M* = 21.98, *SD* = 7.05), *F*(1, 335) = .09, *p* = .77. Figure 1 outlines the progression and associated drop-out rates. It is notable that there was an 8% drop-out at the outset of the general knowledge questions (i.e., question 10). The attrition reached 20% at questions 49 (of 56 questions); with full questionnaire attrition equating to 20.89%. Given that respondent fatigue can impact completion (see, e.g., Hochheimer et al., 2016), the number of questions is, not surprisingly, a potential factor in partial completion of surveys. Visual inspection of attrition rates across the questionnaire suggests that the length may have been a factor in overall attrition.

**FIGURE 1**

Attrition across the questionnaire as indicated by participant drop-out.

Analyses were conducted using chi-square analyses, and were interpreted according to conventions for the data (e.g., using Yates’ Correction for Continuity; Monte Carlo methods). While there were a small number of significant findings (*n* = 13, of the 94 items), these results indicated there was not a pattern to suggest a meaningful difference between completers and those who did not complete the questionnaire. For example, although one symptom (i.e., the affected part of the body from gluten is the small intestine) was correctly identified by a larger proportion of those participants who left the study (89%, compared with 65%), there were no significant differences for the other symptoms which preceded and followed this question.

## **Analyses to compare participant survey completers and non-completers**

**TABLE 2**

### Chi-square analyses to compare participant completers versus non-completers.

| **Questions** | ***df*** | ***n*** | | **Chi-square** | | ***p*** | | **Effect size** | |
| --- | --- | --- | --- | --- | --- | --- | --- | --- | --- |
| 1. Have you heard of coeliac disease? | 1 | 330 | | .00 | | .96 | | *phi* = .02 | |
| 2. How many people do you think are affected by coeliac disease? | 5 | 324 | | 6.34 | | .27 | | *Cramer’s V* = .14 (Monte Carlo) | |
| 3. How many people do you think are affected by peanut allergy? | 5 | 324 | | 5.15 | | .40 | | *Cramer’s V* = .13 (Monte Carlo) | |
| 4. What is coeliac disease? | 4 | 321 | | 5.05 | | .27 | | *Cramer’s V* = .13 (Monte Carlo) | |
| 5. What causes coeliac disease? | 3 | 321 | | 4.63 | | .21 | | *Cramer’s V* = .12 (Monte Carlo) | |
| 6. For what symptoms and signs can you suspect the presence of coeliac disease in an adult? | | | | | | | | | |
| Chronic diarrhea or constipation*** | 1 | 318 | | 14.70 | | < .001 | | *phi* = .22 | |
| Weight deficiency* | 1 | 318 | | 6.64 | | .01 | | *phi* = .16 | |
| Iron deficiency anaemia for unknown reasons | 1 | 318 | | 3.42 | | .10 | | *phi* = .10 | |
| Frequent abdominal pain and bloating* | 1 | 318 | | 5.34 | | .02 | | *phi* = .15 | |
| Short stature | 1 | 318 | | .68 | | .41 | | *phi* = .06 | |
| Osteoporosis | 1 | 318 | | .80 | | .37 | | *phi* = .06 | |
| Presence of irritable bowel syndrome** | 1 | 318 | | 7.93 | | < .01 | | *phi* = .16 | |
| The presence of chronic fatigue syndrome | 1 | 318 | | .21 | | .64 | | *phi* = .04 | |
| Elevated hepatic ALT and AST for unknown reasons | 1 | 318 | | .19 | | .66 | | *phi* = .04 | |
| No apparent symptoms. | 1 | 318 | | .38 | | .54 | | *phi* = .06 | |
| 7. For what symptoms and signs can you suspect the presence of coeliac disease in a child? | | | | | | | | | |
| Chronic diarrhea or constipation*** | 1 | 316 | | 14.36 | | < .001 | | *phi* = .23 | |
| Frequent abdominal pain*** | 1 | 316 | | 15.81 | | < .001 | | *phi* = .24 | |
| Big belly | 1 | 316 | | 2.95 | | .09 | | *phi* = .11 | |
| Vomiting** | 1 | 316 | | 8.47 | | .004 | | *phi* = .18 | |
| Weight deficiency, decreased muscle mass* | 1 | 316 | | 6.06 | | .01 | | *phi* = .15 | |
| Poor appetite** | 1 | 316 | | 9.03 | | .003 | | *phi* = .18 | |
| Short stature | 1 | 316 | | 1.11 | | .29 | | *phi* = .07 | |
| Irritability, tearfulness** | 1 | 316 | | 9.71 | | .002 | | *phi* = .19 | |
| Iron deficiency anemia for unknown reasons*** | 1 | 316 | | 11.35 | | < .001 | | *phi* = .20 | |
| Frequent colds | 1 | 316 | | 2.24 | | .13 | | *phi* = .10 | |
| Sometimes no apparent symptoms. | 1 | 316 | | .88 | | .35 | | *phi* = .06 | |
| 8. Which of the following diseases can be associated with coeliac disease? | | | | | | | | | |
| Delayed sexual development in children | 1 | 315 | | .10 | | .75 | | *phi* = .03 | |
| Infertility | 1 | 315 | | .94 | | .33 | | *phi* = .07 | |
| Osteopenia, osteoporosis | 1 | 317 | | 1.67 | | .20 | | *phi* = .08 | |
| Immunoglobulin A deficiency | 1 | 315 | | .77 | | .38 | | *phi* = .06 | |
| Hypoplasia of tooth enamel | 1 | 315 | | .06 | | .81 | | *phi* = .03 | |
| Recurrent aphthous stomatitis | 1 | 315 | | .61 | | .44 | | *phi* = .06 | |
| Type 1 diabetes | 1 | 315 | | .20 | | .65 | | *phi* = -.04 | |
| Autoimmune thyroiditis | 1 | 316 | | 1.43 | | .23 | | *phi* = .08 | |
| Autoimmune gastritis (pernicious anemia) | 1 | 316 | | 3.76 | | .052 | | *phi* = .12 | |
| Herpetiformis dermatitis, psoriasis | 1 | 315 | | 2.05 | | .15 | | *phi* = .09 | |
| Down syndrome, Turner syndrome | 1 | 315 | | .00 | | .97 | | *phi* = .02 | |
| Peripheral neuropathy, ataxia, epilepsy | 1 | 315 | | .00 | | 1.00 | | *phi* = .01 | |
| 9. Is it recommended that close relatives of patients with coeliac disease be examined for coeliac disease? | 1 | 299 | | .27 | | .60 | | *phi* = .03 | |
| 10. Coeliac disease is an auto-immune disease | 2 | 320 | | .48 | | .80 | | *Cramer’s V* = .04 (Monte Carlo) | |
| 11. Coeliac disease is | 2 | 317 | | .17 | | .94 | | *Cramer’s V* = .02 (Monte Carlo) | |
| 12. Coeliac disease is a genetic disease | 2 | 317 | | 1.60 | | .45 | | *Cramer’s V* = .07 | |
| 13. The available treatment for coeliac disease | 3 | 317 | | 3.91 | | .21 | | *Cramer’s V* = .11 (Monte Carlo) | |
| 14. The affected part of body from gluten* | 2 | 310 | | 6.06 | | < .05 | | *Cramer’s V* = .14 (Monte Carlo) | |
| 15. The gluten-free diet is | 2 | 310 | | .93 | | .77 | | *Cramer’s V* = .06 (Monte Carlo) | |
| 16. I don’t need to see a doctor or a registered dietitian if I‘m following the gluten free diet | 1 | 310 | | .39 | | .53 | | *phi* = .04 | |
| 17. Can coeliac disease be cured? | 2 | 304 | | .44 | | 1.00 | | *Cramer’s V* = .04 (Monte Carlo) | |
| 18. What is gluten? | 3 | 304 | | .29 | | 1.00 | | *Cramer’s V* = .03 (Monte Carlo) | |
| 19. You are buying tomato sauce. How can you find out if it contains gluten? | 3 | 293 | | .40 | | 1.00 | | *Cramer’s V* = .04 (Monte Carlo) | |
| 20. Please decide if the following foods are Foods Allowed, Foods to Question, or Foods Not Allowed in a gluten-free diet. | | | | | | | | | |
| Cocoa | 2 | 297 | | 1.83 | | .40 | | *Cramer’s V* = .08 (Monte Carlo) | |
| Malt vinegar | 2 | | 297 | | .30 | | .88 | | *Cramer’s V* = .03 (Monte Carlo) |
| Croutons | 2 | | 297 | | 6.12 | | .06 | | *Cramer’s V* = .14 (Monte Carlo) |
| Flavored yoghurt | 2 | | 297 | | 1.18 | | .76 | | *Cramer’s V* = .06 (Monte Carlo) |
| Sausages | 2 | | 297 | | .57 | | .83 | | *Cramer’s V* = .04 (Monte Carlo) |
| Imitation seafood | 2 | | 297 | | 3.31 | | .23 | | *Cramer’s V* = .04 (Monte Carlo) |
| Balsamic vinegar | 2 | | 297 | | .73 | | .83 | | *Cramer’s V* = .05 (Monte Carlo) |
| Chickpea flour | 2 | | 297 | | 1.31 | | .55 | | *Cramer’s V* = .07 (Monte Carlo) |
| Glutinous rice | 2 | | 297 | | .26 | | .92 | | *Cramer’s V* = .03 (Monte Carlo) |
| Rice crisp cereal** | 2 | | 297 | | 9.57 | | .01 | | *Cramer’s V* = .18 (Monte Carlo) |
| Soy sauce | 2 | | 297 | | 3.34 | | .20 | | *Cramer’s V* = .11 (Monte Carlo) |
| Milk | 2 | | 297 | | .72 | | .79 | | *Cramer’s V* = .05 (Monte Carlo) |
| Buckwheat | 2 | | 297 | | 1.07 | | .59 | | *Cramer’s V* = .06 (Monte Carlo) |
| Modified corn starch | 2 | | 297 | | .85 | | .66 | | *Cramer’s V* = .05 (Monte Carlo) |
| Spelt | 2 | | 297 | | .58 | | .78 | | *Cramer’s V* = .04 (Monte Carlo) |
| Egg noodles | 2 | | 297 | | 1.71 | | .44 | | *Cramer’s V* = .08 (Monte Carlo) |
| Oatmeal | 2 | | 297 | | 1.04 | | .64 | | *Cramer’s V* = .06 (Monte Carlo) |
| 22. Gluten is present in which of the following? | 4 | | 299 | | 4.26 | | .29 | | *Cramer’s V* = .12 (Monte Carlo) |
| 23. All fresh fruits are safe to eat by someone on a gluten-free diet. | 1 | | 299 | | .08 | | .78 | | *phi* = -.02 |
| 24. It is safe to use the same gloves to touch gluten-containing and gluten-free food items because the amount of cross contamination from this practice is so small that no harm will happen to someone on a gluten-free diet. | 1 | | 289 | | .30 | | .59 | | *phi* = -.03 |
| 25. Soy sauce needs to be added to a gluten-free dish. Which of the following is the right strategy? | 2 | | 286 | | .45 | | .80 | | *Cramer’s V* = .04 (Monte Carlo) |
| 26. While making a gluten-free meal, it is safe to use boiling water for gluten-free pasta if it has been already used to cook wheat pasta as long as all visible bits of wheat pasta are carefully removed by running the water through a sieve/colander. | 1 | | 286 | | .20 | | .65 | | *phi* = -.03 |
| 27. Cheesecake is being served for dessert at dinner. Which of the following would you tell a patient on gluten free diet? | 1 | | 285 | | .09 | | .77 | | *phi* = .02 |
| 28. Spaghetti sauce is being added while cooking a gluten-free meal. It is a famous imported brand. The ingredient list on the bottle mentions protein flour, but no gluten-containing grains. Which of the following is the correct strategy? | 2 | | 285 | | .17 | | 1.00 | | *Cramer’s V* = .02 (Monte Carlo) |
| 29. A frying pan is used to make breaded fish. If gluten-free breaded fish is to be made later, what would be the correct strategy? | 2 | | 285 | | .08 | | 1.00 | | *Cramer’s V* = .02 (Monte Carlo) |
| 30. A grill is being used to cook seasoned chicken breast. The regular seasoning recipe has gluten. You need to cook a chicken breast without seasoning for a patient on a gluten-free diet. How would you cook this chicken? | 2 | | 285 | | .06 | | .97 | | *Cramer’s V* = .01 (Monte Carlo) |
| 31. Pizzas were made for lunch. There are regular pizzas and gluten-free pizzas. What is the correct strategy to cut the slices? | 2 | | 285 | | .09 | | 1.00 | | *Cramer’s V* = .02 (Monte Carlo) |
| 32. You are cutting up slices of bread (both gluten-containing and gluten-free bread) on a cutting board. What is the correct strategy? | 2 | | 285 | | .25 | | 1.00 | | *Cramer’s V* = .03 (Monte Carlo) |
| 33. A person with coeliac disease asks for salad dressing for their salad. You have only Italian salad dressing. You read the label that lists the following: | 2 | | 293 | | .04 | | .84 | | *Cramer’s V* = .01 (Monte Carlo) |
| 34. A person on a gluten-free diet wants to have a muffin. There is some pure millet flour and some oat flour in the kitchen. Which flour(s) are gluten free and safe to use to make the muffin? | 3 | | 293 | | 2.91 | | .41 | | *Cramer’s V* = .10 (Monte Carlo) |
| 35. Ice cream is being served to a person on a gluten-free diet. Which is the correct statement? | 2 | | 289 | | .75 | | 1.00 | | *Cramer’s V* = .05 (Monte Carlo) |
| 36. If you accidently put croutons in the salad, to make it safe for the person with coeliac disease, would you: | 3 | | 293 | | 1.18 | | .79 | | *Cramer’s V* = .06 (Monte Carlo) |
| 37. A toaster oven in the kitchen is used to toast slices of bread for breakfast. Which of the following is the safest method for toasting gluten-free bread? | 2 | | 289 | | .56 | | 1.00 | | *Cramer’s V* = .04 (Monte Carlo) |
| 38. If you were making a burger for a person with coeliac disease and accidentally put a gluten-free beef burger patty, lettuce, tomato, pickles and tomato sauce on an ordinary burger bun, would you: | 2 | | 293 | | .67 | | .87 | | *Cramer’s V = .05 (Monte Carlo)* |
| 39. Quinoa is for dinner today instead of rice. Which of the following is true? | 2 | | 286 | | .62 | | 1.00 | | *Cramer’s V = .05 (Monte Carlo)* |
| 40. After cooking a beef burger patty and placing it on a gluten-free bun for a person with coeliac disease, you discover that someone had just finished using the same frying pan to cook a toasted cheese sandwich. | 2 | | 293 | | .44 | | 1.00 | | *Cramer’s V = .04 (Monte Carlo)* |
| 41. If you were making a meal for a coworker with coeliac disease, which of the following would you regard as essential, nice but non-essential, unnecessary in preparing their food. | | | | | | | | | |
| Opening a new container of peanut butter | 2 | 295 | | .04 | | 1.00 | | *Cramer’s V* = .01 (Monte Carlo) | |
| Keep all cheese separate from the food preparation area so it does not come in contact with gluten free food | 2 | 295 | | 1.78 | | .43 | | *Cramer’s V* = .08 (Monte Carlo) | |
| Use a deep fryer that is used only for gluten-free foods | 2 | 295 | | .37 | | 1.00 | | *Cramer’s V* = .04 (Monte Carlo) | |
| Wash your hands or change your gloves after touching gluten-containing food | 2 | 295 | | 3.69 | | .17 | | *Cramer’s V* = .11 (Monte Carlo) | |
| Make the food in a dedicated kitchen in which you prepare only gluten-free food | 2 | 295 | | 4.89 | | .09 | | *Cramer’s V* = .13 (Monte Carlo) | |
| Avoid cutting a gluten free sandwich with a knife you used to cut an ordinary sandwich | 2 | 295 | | .04 | | 1.00 | | *Cramer’s V* = .01 (Monte Carlo) | |
| Avoid using an ordinary toaster to toast gluten-free bread | 2 | 295 | | .50 | | 1.00 | | *Cramer’s V* = .04 (Monte Carlo) | |
| Avoid cooking the gluten free pasta in the water that was used to cook the ordinary pasta | 2 | 295 | | .13 | | 1.00 | | *Cramer’s V* = .02 (Monte Carlo) | |

**p* < .05

** p < .01

*** p < .001

|  |
| --- |

## **Correlations between individual items and the full 41-item scale**

**TABLE 3**

### Point-biserial correlation results for the AKCD (Alorayyidh et al., 2023) questions included in the 41-item scale

|  |  | Results |  |
| --- | --- | --- | --- |
| Scale Items | *N* | *rpb* | *p* |

|  |  |  |  |
| --- | --- | --- | --- |
| Coeliac disease is an auto-immune disease*** | 284 | .339 | < .001 |
| Coeliac disease is *chronic**** | 284 | .456 | < .001 |
| Coeliac disease is a genetic disease*** | 284 | . 421 | < .001 |
| The affected part of the body from gluten*** | 284 | .344 | < .001 |
| The available treatment for coeliac disease*** | 284 | .313 | < .001 |
| The gluten free diet is *lifelong**** | 284 | .315 | < .001 |
| Need to see a doctor or registered dietician* | 284 | .146 | .014 |

**p* < .05

** p < .01

*** p < .001

**TABLE 4**

### Point-biserial correlation results for the questions from Kozhakhmetova et al. (2022) included in the 41-item scale

|  |  | Results |  |
| --- | --- | --- | --- |
| Scale Items | *N* | *rpb* | *p* |

| What is coeliac disease?*** | 284 | .359 | < .001 |
| --- | --- | --- | --- |
| What causes coeliac disease? | 284 | -.022 | .717 |
| Is it recommended that close relatives of patients with coeliac disease be examined for coeliac disease? *** | 284 | .196 | < .001 |
| *Symptoms and signs that indicate the presence of coeliac disease in adults* | | | |
| Chronic diarrhea or constipation*** | 284 | .416 | < .001 |
| Weight deficiency*** | 284 | .531 | < .001 |
| Iron deficiency anaemia*** | 284 | .594 | < .001 |
| Frequent abdominal pain and bloating*** | 284 | .540 | < .001 |
| Short stature*** | 284 | .381 | < .001 |
| Osteoporosis*** | 284 | .496 | < .001 |
| Presence of irritable bowel syndrome*** | 284 | .357 | < .001 |
| The presence of chronic fatigue syndrome*** | 284 | .510 | < .001 |
| Elevated hepatic ALT and AST *** | 284 | .499 | < .001 |
| No apparent symptoms*** | 284 | .263 | < .001 |
| *Symptoms and signs that indicate the presence of coeliac disease in children* | | | |
| Chronic diarrhea or constipation*** | 284 | .439 | < .001 |
| Frequent abdominal pain*** | 284 | .566 | < .001 |
| Big belly*** | 284 | .501 | < .001 |
| Vomiting*** | 284 | .419 | < .001 |
| Weight deficiency, decreased muscle mass*** | 284 | .514 | < .001 |
| Poor appetite*** | 284 | .474 | < .001 |
| Short stature*** | 284 | .462 | < .001 |
| Irritability, tearfulness*** | 284 | .520 | < .001 |
| Iron deficiency anemia for unknown reasons*** | 284 | .608 | < .001 |
| Frequent colds*** | 284 | .485 | < .001 |
| Sometimes no apparent symptoms*** | 284 | .418 | < .001 |
| *Which of the following diseases can be associated with coeliac2 disease (tick all that apply)?* | | | |
| Delayed sexual development in children*** | 284 | .418 | < .001 |
| Infertility*** | 284 | .502 | < .001 |
| Osteopenia, osteoporosis*** | 284 | .544 | < .001 |
| Immunoglobulin A deficiency*** | 284 | .487 | < .001 |
| Hypoplasia of tooth enamel*** | 284 | .463 | < .001 |
| Recurrent aphthous stomatitis*** | 284 | .422 | < .001 |
| Type 1 diabetes*** | 284 | .439 | < .001 |
| Autoimmune thyroiditis*** | 284 | .491 | < .001 |
| Autoimmune gastritis (pernicious anemia) *** | 284 | .479 | < .001 |
| Herpetiformis dermatitis, psoriasis*** | 284 | .532 | < .001 |
| Down syndrome, Turner syndrome*** | 284 | .324 | < .001 |
| Peripheral neuropathy, ataxia, epilepsy*** | 284 | .401 | < .001 |

**p* < .05

** p < .01

*** p < .001

**TABLE 5**

### Point-biserial correlation results for the GFD-KS (Silvester et al., 2016) questions included in the 41-item scale

|  |  | Results |  |
| --- | --- | --- | --- |
| Scale Items | *N* | *rpb* | *p* |

| Cocoa | 284 | .112 | .059 |
| --- | --- | --- | --- |
| Malt vinegar*** | 284 | .350 | < .001 |
| Croutons | 284 | -.022 | .706 |
| Flavored yoghurt*** | 284 | .296 | < .001 |
| Sausages*** | 284 | .377 | < .001 |
| Imitation seafood*** | 284 | .281 | < .001 |
| Balsamic vinegar | 284 | .071 | .233 |
| Chickpea flour*** | 284 | .340 | < .001 |
| Glutinous rice*** | 284 | .338 | < .001 |
| Rice crisp cereal*** | 284 | .205 | < .001 |
| Soy sauce* | 284 | .141 | .018 |
| Milk*** | 284 | .267 | < .001 |
| Buckwheat*** | 284 | .428 | < .001 |
| Modified corn starch*** | 284 | .268 | < .001 |
| Spelt*** | 284 | .276 | < .001 |
| Egg noodles* | 284 | .135 | .023 |
| Oatmeal | 284 | .079 | .183 |

**p* < .05

** p < .01

*** p < .001

**TABLE 6**

### Point-biserial correlation results for the questions from Zhou et al. (2022) included in the 41-item scale

|  |  | Results |  |
| --- | --- | --- | --- |
| Scale Items | *N* | *rpb* | *p* |

| Opening a new container of peanut butter*** | 284 | .305 | < .001 |
| --- | --- | --- | --- |
| Keep all cheese separate from the food preparation area so it does not come in contact with gluten free food | 284 | -.061 | .309 |
| Use a deep fryer that has is used only for gluten-free foods*** | 284 | .214 | < .001 |
| Wash your hands or change your gloves after touching gluten-containing food* | 284 | .150 | .011 |
| Make the food in a dedicated kitchen in which you prepare only gluten-free food* | 284 | .146 | .014 |
| Avoid cutting a gluten free sandwich with a knife you used to cut an ordinary sandwich*** | 284 | .333 | < .001 |
| Avoid using an ordinary toaster to toast gluten-free bread*** | 284 | -.224 | < .001 |
| Avoid cooking the gluten free pasta in the water that was used to cook the ordinary pasta*** | 284 | .221 | < .001 |
| Gluten belongs to which of the following nutrient groups? | 284 | .180 | .002 |
| Gluten is present in which of the following? | 284 | .282 | < .001 |
| All fresh fruits are safe to eat by someone on a gluten-free diet. | 284 | .093 | .119 |
| If you accidently put croutons in the salad, to make it safe for the person with coeliac disease, would you: *** | 284 | .324 | < .001 |
| After cooking a beef burger patty and placing it on a gluten-free bun for a person with coeliac disease, you discover that someone had just finished using the same frying pan to cook a toasted cheese sandwich. Do you: *** | 284 | .250 | < .001 |
| If you were making a burger for a person with coeliac disease and accidentally put a gluten-free beef burger patty, lettuce, tomato, pickles and tomato sauce on an ordinary burger bun, would you: *** | 284 | .357 | < .001 |
| A person with coeliac disease asks for salad dressing for their salad. … It is safe to serve this salad dressing to the person? *** | 284 | .286 | < .001 |
| A person on a gluten-free diet wants to have a muffin. … Which flour(s) are gluten free and safe to use to make the muffin? *** | 284 | .235 | < .001 |
| Ice cream is being served to a person on a gluten-free diet. Which is the correct statement? *** | 284 | .320 | < .001 |
| A toaster oven in the kitchen is used to toast slices of bread for breakfast. Which of the following is the safest method for toasting gluten-free bread? *** | 284 | .251 | < .001 |
| It is safe to use the same gloves to touch gluten-containing and gluten-free food items because the amount of cross contamination from this practice is so small that no harm will happen to someone on a gluten-free diet. *** | 284 | .351 | < .001 |
| Quinoa is for dinner today instead of rice. Which of the following is true? *** | 284 | .240 | < .001 |
| Soy sauce needs to be added to a gluten-free dish. Which of the following is the right strategy? *** | 284 | .220 | < .001 |
| While making a gluten-free meal, it is safe to use boiling water for gluten-free pasta if it has been already used to cook wheat pasta as long as all visible bits of wheat pasta are carefully removed by running the water through a sieve/colander. *** | 284 | .320 | < .001 |
| Cheesecake is being served for dessert at dinner. Which of the following would you tell a patient on gluten free diet? *** | 284 | .305 | < .001 |
| Spaghetti sauce is being added while cooking a gluten-free meal. … Which of the following is the correct strategy? *** | 284 | .351 | < .001 |
| A frying pan is used to make breaded fish. If gluten-free breaded fish is to be made later, what would be the correct strategy? *** | 284 | .296 | < .001 |
| A grill is being used to cook seasoned chicken breast. … How would you cook this chicken? *** | 284 | .266 | < .001 |
| Pizzas were made for lunch. … What is the correct strategy to cut the slices? *** | 284 | .392 | < .001 |
| You are cutting up slices of bread (both gluten-containing and gluten-free bread) on a cutting board. What is the correct strategy? *** | 284 | .289 | < .001 |

**p* < .05

** p < .01

*** p < .001

**TABLE 7**

### Point-biserial correlation results for the additional questions from previous research included in the 41-item scale

|  |  | Results |  |
| --- | --- | --- | --- |
| Scale Items | *N* | *rpb* | *p* |

| You are buying tomato ketchup. How can you find out if it contains gluten?** | 284 | .162 | .006 |
| --- | --- | --- | --- |
| Have you heard of coeliac disease?*** | 284 | .342 | < .001 |
| How many people do you think are affected by coeliac disease?** | 284 | .170 | .004 |
| How many people do you think are affected by peanut allergy? | 284 | .102 | .087 |
| Can coeliac disease be cured?*** | 284 | .450 | < .001 |
| What is gluten?*** | 284 | .231 | < .001 |

**p* < .05

** p < .01

*** p < .001
